# Clinically Correlated MicroRNAs in the Diagnosis of Non-Small Cell Lung Cancer: A Systematic Review and Meta-Analysis

**DOI:** 10.1155/2018/5930951

**Published:** 2018-06-28

**Authors:** Min Jiang, Xuelian Li, Xiaowei Quan, Xiaoying Li, Baosen Zhou

**Affiliations:** ^1^Department of Clinical Epidemiology and Center of Evidence-Based Medicine, The First Affiliated Hospital, China Medical University, Shenyang, Liaoning 110001, China; ^2^Department of Epidemiology, School of Public Health, China Medical University, Shenyang 110122, China

## Abstract

*(1) Background*. Non-small cell lung cancer (NSCLC) has a high mortality rate. MiRNAs have been found to be diagnostic biomarkers for NSCLC. However, controversial results exist. We conducted this meta-analysis to evaluate the diagnostic value of miRNAs for NSCLC.* (2) Methods*. Databases and reference lists were searched. Pooled sensitivity (SEN), specificity (SPE), and area under the curve (AUC) were applied to examine the general diagnostic efficacy, and subgroup analysis was also performed.* (3) Results*. Pooled SEN, SPE, and AUC were 85%, 88%, and 0.93, respectively, for 71 studies. Multiple miRNAs (AUC: 0.96) obtained higher diagnostic value than single miRNA (AUC: 0.86), and the same result was found for Caucasian population (AUC: 0.97) when compared with Asian (AUC: 0.91) and Caucasian/African population (AUC: 0.92). MiRNA had higher diagnostic efficacy when participants contained both smokers and nonsmokers (AUC is 0.95 for imbalanced group and 0.91 for balanced group) than when containing only smokers (AUC: 0.90). Meanwhile, AUC was 0.91 for both miR-21 and miR-210.* (4) Conclusions*. Multiple miRNAs such as miR-21 and miR-210 could be used as diagnostic tools for NSCLC, especially for the Caucasian and nonsmoking NSCLC.

## 1. Introduction

Lung cancer is the principal cause of cancer-associated deaths among males both in developed and in developing countries, and it has exceeded the breast cancer becoming the major cause of cancer-related deaths in females in the developed countries [1]. Non-small cell lung cancer (NSCLC) is a major type of lung cancer that is responsible for 85% lung cancer-associated deaths. Smoking has been recognized as a primary environmental risk factor of lung cancer. However, only a small number of smokers will develop into lung cancer patients.

MicroRNA is a group of 19–22 nucleotide, small, single-stranded, and conserved noncoding RNA that acts as a regulator of gene expression at both the posttranscriptional and the translational levels through acting on the 3′-untranslated region (UTR) of messenger RNA (mRNA) [2]. MiRNAs play important roles in various biological processes associated with the tumorigenesis such as the cellular proliferation, differentiation, metabolism, and apoptosis [3, 4]. It is available to isolate the miRNAs from the clinical specimens including the plasma, serum, sputum, and tissue. Meanwhile, it has a high stability. Due to these advantages, the miRNAs are increasingly becoming an ideal tool for the detection of NSCLC.

Recently, a series of articles have shown that different miRNAs might be applied to detect the NSCLC [5–7]. For example, miR-21, an oncogenic miRNA, has been shown to be overexpressed in lung cancer as well as other various human tumors [8]. Upregulation of miR-21 could promote the tumorigenesis of lung cancer through inhibiting the apoptosis process and negatively regulating the Ras/MEK/ERK signal pathway [9]. High miR-210 expression was correlated with the increased lymph node metastasis and a poor prognosis in patients with NSCLC [10]. Both these two, miR-21 and miR-210, have been explored to be used as diagnostic tools for NSCLC, no matter whether they are applied in combination with other miRNAs or alone [11–14]. However, as a result of the small sample sizes, the different miRNAs profiling, and the differences of the specimen and ethnicity, inconsistencies existed among studies that had examined the diagnostic value of miR-21, miR-210, and other miRNAs for NSCLC. Therefore, a meta-analysis was performed to assess the performance of miRNAs in the detection for NSCLC.

## 2. Materials and Methods

### 2.1. Search Strategy

Our meta-analysis was based on the Preferred Reporting Items for Systematic Reviews and Meta-Analyses (PRISMA). We searched PubMed, Google Scholar, Chinese National Knowledge Infrastructure (CNKI), Embase, and Medline to find all associated articles in order to investigate the potential utility of miRNAs as diagnostic tools for NSCLC. The combination of the Medical Subject Headings (MeSH) and the keywords (“lung neoplasm” OR “lung malignancy” OR “lung cancer”) AND (“miRNA” OR “microRNAs”) AND (“ROC curve” OR “sensitivity” OR “specificity” OR “diagnosis”) was used (updated to April 5, 2017). The reference lists of the reviews were also searched to obtain all the acceptable articles.

### 2.2. Study Selection

A series of criteria were applied for study inclusion and exclusion. For inclusion, the criteria were as follows: (1) patients with NSCLC; (2) the type of the controls being healthy controls (HC) or patients with benign pulmonary diseases (BPD); (3) assessing the diagnostic value of the miRNAs; (4) the possibility of extracting or calculating TP, FP, FN, and TN from the articles. For exclusion, the criteria were as follows: (1) studies that were duplicate publications, reviews, or unrelated; (2) studies without complete data.

### 2.3. Data Collection and Quality Assessment

Two authors collected the data independently as follows: the first author, publication year, and participant demographic characteristics (ethnicity, sample size, mean or median age, smoking status, the types of the controls, and the testing method of controls and cancer); types of the specimen; miRNA profiling and the data used for this meta-analysis (SEN, SPE, TP, FP, FN, and TN). The quality of these articles were assessed with the QUADAS-2 guidelines [15].

### 2.4. Statistical Analysis

All the statistical analyses were conducted by RevMan 5.3 (version 1.4) software and STATA 11.0 (STATA-Corp, College Station, TX, version 11.0) software. The heterogeneity among the selected studies was assessed through the Q test and the I^2^ value [16]. The* P* value for the Q test being less than 0.05 or the I^2^ ≥ 50 % demonstrated that there was heterogeneity among the included studies. The pooled SPE [TN/(FP+TN)], SEN [TP/(FN+TP)], diagnostic odds ratio (DOR) [PLR/NLR], the negative likelihood ratio (NLR) [(1-SPE)/SPE)], the positive likelihood ratio (PLR) [(SEN/(1-SEN)], and their 95% confidence intervals (95% CIs) were evaluated by a bivariate random-effect-regression model. The SROC curve was constructed and the AUC value was calculated too. A Fagan nomogram was also constructed to evaluate the clinical utility of miRNAs in the diagnosis of NSCLC. Subgroup analyses (grouped by miRNA profiling: single and multiple; smoking status: only smokers, smokers, and nonsmokers (imbalanced between groups), smokers and nonsmokers (balanced between groups), and unknown smoking status; specimen: serum, plasma, whole blood/blood cell, and not blood; ethnicity: Asian, Caucasian, and Caucasian/African; control-type: BPD, HC, and BPD/HC; stage: early stage and no early stage; and case number: large (≥ 50) and small (< 50)) and meta-regression analysis were used to identify the potential sources of the heterogeneity. The Deeks' funnel plot asymmetry test was also applied to explore the publication bias, with the* P *value less than 0.01 considered significant [17].

## 3. Results

### 3.1. Literature Search and the Studies' Characteristics

**Figure 1:**
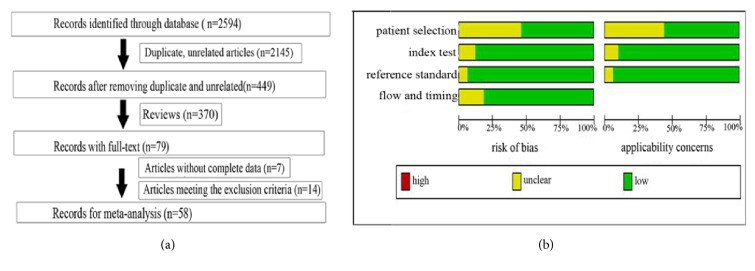
Flow chart of this meta-analysis of miRNAs in NSCLC detection (**a**) and the quality of these included articles according to the QUADAS-2 guidelines: proportion of articles with risk of bias (left) and proportion of articles with concerns regarding applicability (right) (**b**).

As shown in Figure 1(a), 2594 eligible articles were included, of which 2145 articles were removed as unrelated and duplicate articles. And then 370 reviews were also excluded, leaving 79 articles with full texts, and another 21 articles were then removed through carefully reading: 14 articles met the exclusion criteria and 7 articles did not have the complete data. Ultimately, 58 articles [5–7, 11–14, 18–68] with 71 studies published from 2009 to 2017 including 9,099 participants (5111 cases with NSCLC and 3988 controls from the healthy individuals and the patients with the benign pulmonary disease (BPD)) were included. The main characteristics of these 71 studies were shown in Table 1. Wang Y's article [7], Fan LH's article [52], Nadal E's article [45], Tang DF's article [32], Razzak R's article [14], Wang W's article [68], Yu L's article [19], and Xing LX's article [18] included 2 studies. Bediaga's article [28] included 3 studies, Wang C's article [46] included 4 studies, and the remaining articles [5, 6, 11–13, 20–27, 29–31, 33–44, 47–51, 53–67] included 1 study, respectively. Meanwhile, there were 18 studies [13, 14, 28, 31, 33, 45, 46, 48, 54, 56, 63, 65, 66] performed in Caucasian, 11 studies [18, 19, 21, 23, 27, 29, 30, 38, 44] performed in Caucasian/African, and 1 study [26] performed in African populations; the remaining studies were performed in Asian populations. A total of 50 studies detected the miRNAs in blood such as the whole blood, plasma, serum, and peripheral blood mononuclear cells (PBMC) [6, 7, 11, 20–24, 26, 29–35, 37, 39–42, 44–47, 49–54, 56–64, 66–68], while the remaining studies were detected in nonblood samples (7 tissue [5, 25, 28, 55, 68], 1 pleural effusion [43], 12 sputum [12, 14, 18, 19, 27, 36, 38, 48, 65], and 1 BAL [13]). We evaluated 45 studies for assessing the diagnostic value of multiple miRNAs and 26 studies [5, 6, 11, 20, 22, 24–26, 33–37, 39–41, 43, 47, 50, 51, 55, 57, 58, 60, 67, 68] of single miRNA.

The quantitative real-time polymerase chain reaction (qRT-PCR) and digital polymerase chain reaction (digital PCR) were used in these studies to test the expression levels of different miRNAs, and the most common reference miRNAs were RNU6B, miR-39, and miR-16. Quality of the enrolled studies summarized in Figure 1(b) was generally good.

### 3.2. Pooled Diagnostic Performance

**Figure 2:**
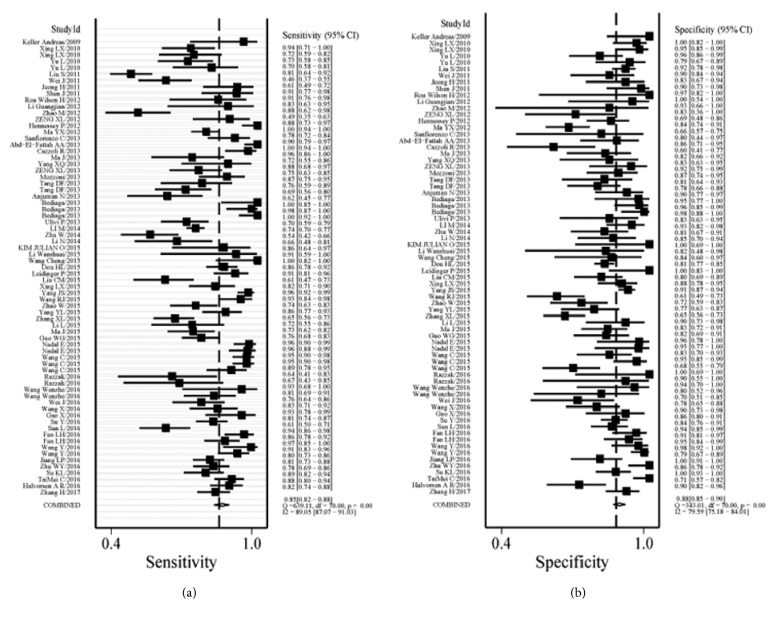
Forest plots of SEN and SPE for the NSCLC diagnosis. Both the SEN and SPE of each study were shown by squares with the 95% confidence interval shown by the error bars.

**Figure 3:**
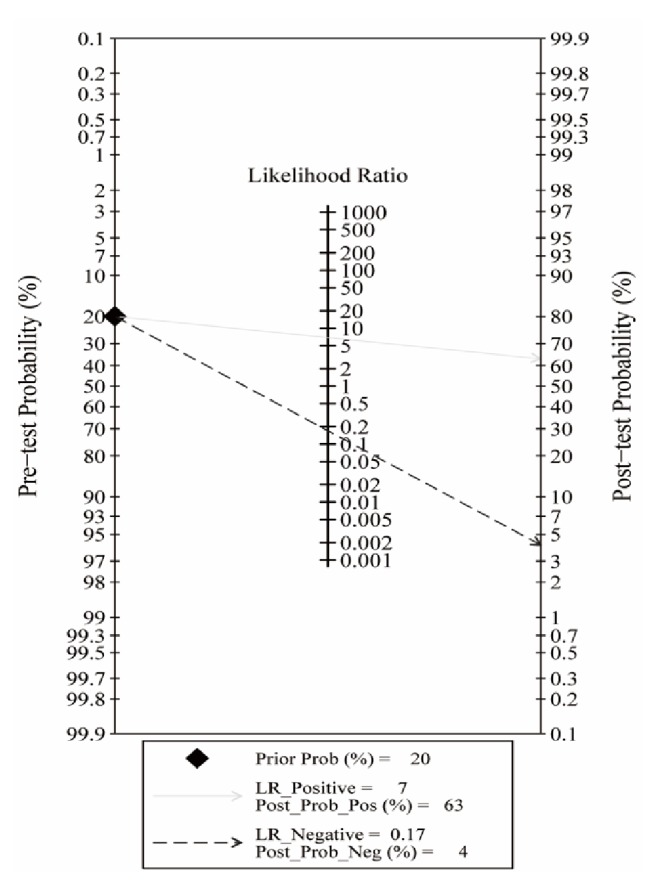
Fagan plot of PLR and NLR to evaluate the clinical utility of miRNAs for diagnosis of NSCLC.

**Figure 4:**
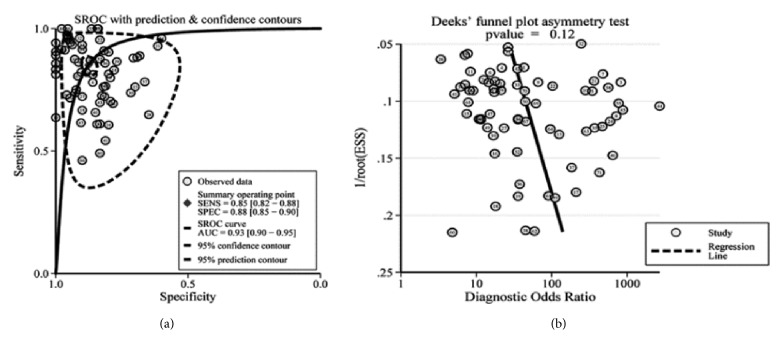
SROC curve of the miRNAs as diagnostic tools for NSCLC (**a**) and the Deeks' test for assessing the publication bias for miRNAs in the detection of NSCLC (**b**).

Significant heterogeneity was obtained since I^2^ values for SEN and SPE were 89.05% (95% CI: 87.07-91.03%) and 79.59% (95% CI: 75.18-84.01%), respectively. Therefore, a random-effect model was conducted for this study. Results indicated the pooled SEN and SPE for these 71 studies were 85% (95% CI: 82-88%) and 88% (95% CI: 85-90%), respectively (Figure 2). The PLR and NLR were 6.9 (95% CI: 5.6-8.4) and 0.17 (95% CI: 0.14-0.21), respectively (Figure 3), the DOR was 40 (95% CI: 28-58), and the AUC was 0.93 (95% CI: 0.90-0.95) (Figure 4(a)).

### 3.3. Publication Bias

Results of the Deeks' funnel plot asymmetry test showed that the publication bias did not exist in these studies as the funnel plot was symmetry (Figure 4(b)) and* P* value equaled 0.12.

### 3.4. Subgroup Analyses and Meta-Regression Analysis

**Figure 5:**
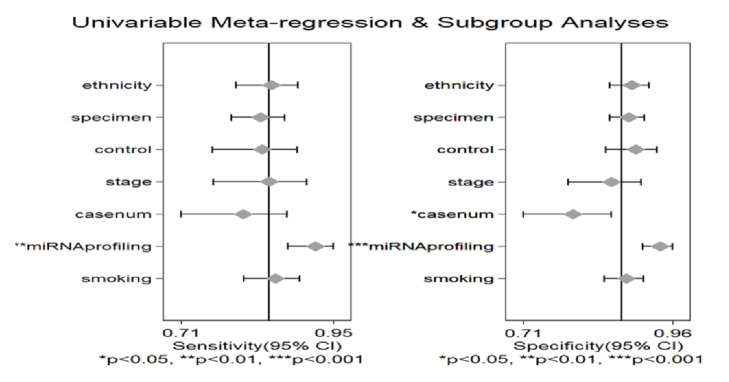
Forest plots for the meta-regression analysis: SEN and SPE. The factors included miRNA profiling, smoking status, specimen, ethnicity, type of control, case number, and stage.

Results of the meta-regression analysis demonstrated that the heterogeneity might be explained by miRNA profiling (*P *< 0.001) and case number (*P *< 0.05) for SPE and by miRNA profiling (*P *< 0.01) for SEN as described in Figure 5. The subgroup analyses were also conducted and the results were presented in Table 2. For the subgroups of smoking status, compared with the subgroup of only smokers (SEN: 80% (95% CI: 70-87%), SPE: 86% (95% CI: 77-91%), and AUC: 0.90 (95% CI: 0.87-0.92)), miRNAs had a higher diagnostic efficacy in the subgroups of smokers and nonsmokers (SEN: 88% (95% CI: 74-95%), SPE: 90% (95% CI: 73-97%), and AUC: 0.95 (95% CI: 0.93-0.97) for imbalanced groups and SEN: 83% (95% CI: 74-90%), SPE: 86% (95% CI: 80-90%), and AUC: 0.91 (95% CI: 0.88-0.93) for balanced groups). Subgroup analysis by specimen showed that studies with serum samples exhibited higher diagnostic accuracy with SEN: 91% (95% CI: 86-95%), SPE: 85% (95% CI: 79-89%), and AUC: 0.94 (95% CI: 0.91-0.95) than studies with plasma samples with the SEN: 82% (95% CI: 76-87%), SPE: 87% (95% CI: 83-90%), and AUC: 0.92 (95% CI: 0.89-0.94) and not blooding samples with the SEN: 80% (95% CI: 72-86%), SPE: 89% (95% CI: 85-93%), and AUC: 0.92 (95% CI: 0.89-0.94), respectively. When compared with the large sample size, miRNA might be a better diagnostic tool for small sample size with SEN: 88% (95% CI: 82-92%), SPE: 91% (95% CI: 88-94%), and AUC: 0.95 (95% CI: 0.93-0.97). In the subgroups for the ethnicity, the miRNAs obtained a better diagnostic value in the Caucasian populations with the SEN: 91% (95% CI: 86-95%), SPE: 92% (95% CI: 87-96%), and AUC: 0.97 (95% CI: 0.95-0.98), respectively, when compared with the Asian populations with the SEN: 82% (95% CI: 77-85%), SPE: 86% (95% CI: 82-88%), and AUC: 0.91 (95% CI: 0.88-0.93), respectively, and the Caucasian/African populations with SEN: 85% (95% CI: 72-93%), SPE: 87% (95% CI: 81-91%), and AUC: 0.92 (95% CI: 0.89-0.94), respectively. In the subgroups of the miRNAs profiling, the multiple miRNAs had a higher accuracy for diagnosing the NSCLC with SEN: 88% (95% CI: 85-91%), SPE: 91% (95% CI: 88-93%), and AUC: 0.96 (95% CI: 0.93-0.97), respectively, when compared with the single miRNA with the SEN: 77% (95% CI: 71-82%), SPE: 80% (95% CI: 77-84%), and AUC: 0.86 (95% CI: 0.82-0.88), respectively. miRNAs had a higher value to distinguish the NSCLC patients from healthy individuals with the SEN: 86% (95% CI: 82-89%), SPE: 88% (95% CI: 85-91%), and AUC: 0.94 (95% CI: 0.91-0.95) than controls with benign pulmonary disease with SEN: 84% (95% CI: 77-89%), SPE: 84% (95% CI: 80-88%), and AUC: 0.90 (95% CI: 0.87-0.92). Compared with other miRNAs, miR-210 and miR-21 were more often used as diagnostic tools. However, they were usually associated with other miRNAs. The sensitivity, specificity, and AUC were, respectively, 77% (95% CI: 72-81%), 93% (95% CI: 88-96%), and 0.91(95% CI: 0.88-0.93) for miR-210 with other miRNAs. The sensitivity, specificity, and AUC of miR-21 with other miRNAs were, respectively, 82% (95% CI: 77-86%), 87% (95% CI: 84-89%), and 0.91 (95% CI: 0.88-0.93).

## 4. Discussion

Due to the high mortality rate and low survival rate of NSCLC, there is an urgent need for the accurate detection method for the early detection of NSCLC especially for the nonsmoking NSCLC patients. Although miRNAs may have a high diagnostic accuracy according to the previous articles, the clinical utility of the miRNA for diagnosing NSCLC remains controversial. Compared with the previous meta-analyses [69–71], there were more studies and participants included in this meta-analysis. Our analysis showed the pooled SEN was 85% (95% CI: 82-88%), the pooled SPE was 88% (95% CI: 85-90%), and the AUC was 0.93 (95% CI: 0.90-0.95), suggesting that miRNAs had pretty high diagnostic value for NSCLC. Our results also showed that the pooled DOR was 40 (95% CI: 28-58), indicating that for an individual proved positive by miRNAs the chance of having NSCLC is 40 times higher than the negative ones. For the subgroup analyses, higher accuracy was observed in the multiple miRNA profiling when compared with the single miRNA, which was consistent with the previous conclusions [69–71]. MiRNAs might have a higher diagnostic efficacy for the nonsmoking NSCLC patients compared with the smoking ones. Meanwhile, differences were also observed among the Caucasian, Asian, and Caucasian/African populations. This result could be supported by the Wang H's article [71]. Furthermore, miRNAs from serum samples exhibited higher diagnostic value than miRNAs from other specimen. These results meant that combinations of various miRNAs may be better diagnostic tools than the single miRNA, and miRNA isolated from serum could have a higher diagnostic value for the Caucasian populations when compared with the Asian and Caucasian/African populations. Among the different multiple miRNAs, miR-210 and miR-21 associated with other miRNAs could be used for the detection of NSCLC. However, there were still some limitations that could not be neglected in this meta-analysis such as the heterogeneity among these 71 studies, the different methods in miRNA profiling, the possibility that some articles are missed or not published online.

## 5. Conclusions

Our meta-analysis showed the practicability of miRNAs for diagnosing NSCLC and demonstrated that the multiple miRNAs might have a relatively high diagnostic value for NSCLC compared with the single miRNA diagnosis. miR-210 and miR-21 could be used as effective tools through combining with other miRNAs. In addition, miRNAs, especially isolated from serum, had a better diagnostic accuracy in Caucasian populations than the Asian populations as well as the Caucasian/African populations. When compared with the smoking NSCLC patients, miRNAs might have a higher diagnostic efficacy for the nonsmoking ones. However, studies on the large samples are still demanded to verify our results.

## Figures and Tables

**Table 1:** The main features of 71 included studies in this meta-analysis.

**Study ID**	**Ethnicity**	**Specimen**	**Case**	**Age**	**Control**	**Age**	**Type of control**	**Stage**	**MiRNA profiling**	**SEN (**%**)**	**SPE (**%**)**	**Reference miRNA**	**microRNA assay**	**Smoking status**_ _^**∗**^
			**N**		**N**									
Zhang H 2017	Asian	plasma	129	59.6	83	60.0	HC	I-II	miR-145, miR-20a, miR-21, miR-223	81.8	90.1	miR-16	qRT-PCR	3
Halvorsen A 2016	Caucasian	serum	100	62.6	58	57.6	HC	I-IV	miR-429, miR-205, miR-200b, miR-203, miR-12, miR-34b	88.0	71.0	miR-220, miR-19b, U6	qRT-PCR	3
TaiMei C2016	Asian	blood	110	65.0	52	65.7	HC	I-III	20 miRNAs_ _^a^	89.1	100	miR-159a, U6	qRT-PCR	4
Su KL 2016	Asian	plasma	100	NA	100	NA	HC	I-III	miR-195	78.0	86.0	miR-39	qRT-PCR	3
Zhu WY 2016	Asian	plasma	112	58.5	40	57.9	HC	I-III	miR-182, miR-183, miR-210, miR-126	81.3	100	U6	qRT-PCR	2
Jiang LP 2016	Asian	tissue	154	54.9	63	57.8	BPD	I-IV	miR-26b	79.9	79.4	U6	qRT-PCR	3
Wang Y 2016	Asian	plasma	82	NA	91	NA	HC	I-II	miR-532, miR-628, miR-425	91.5	97.8	miR-39	qRT-PCR	4
Wang Y 2016	Asian	plasma	36	NA	43	NA	HC	I-II	miR-532, miR-628, miR-425	97.2	95.3	miR-39	qRT-PCR	4
Fan LH 2016	Asian	serum	94	60.5	58	58.1	HC	I-III	miR-15b, miR-16, miR-20a	86.2	91.4	NA	qRT-PCR	4
Fan LH 2016	Asian	serum	70	59.7	54	58.0	HC	I-III	miR-15b, miR-16, miR-20a	94.3	94.2	NA	FQDs	4
Sun L 2016	Asian	plasma	87	60.7	96	53.8	HC,BPD	I-IV	miR-30a	61.0	84.3	U6	qRT-PCR	4
Su Y 2016	Asian	sputum	144	66.3	171	65.2	BPD	I	miR-21, miR-31, miR-210	81.5	85.9	U6	qRT-PCR	1
Gao X 2016	Asian	plasma	30	61.1	30	60.2	HC	I	miR-324, miR-1285	93.3	90.0	miR-39	qRT-PCR	4
Wang X 2016	Asian	plasma	59	55.9	59	57.6	BPD	I-III	miR-486	83.1	78.0	miR-16	qRT-PCR	4
Wei J 2016	Asian	plasma	63	61.0	30	57.0	HC	I-IV	miR-21	76.2	70.0	miR-16	qRT-PCR	3
Razzak 2016	Caucasian	sputum	22	68	10	58	HC,BPD	III-IV	miR-21, miR-210, miR-372	64	100	U6	qRT-PCR	4
Razzak 2016	Caucasian	sputum	21	70	10	58	HC,BPD	I-II	miR-21, miR-210, miR-372	67	90	U6	qRT-PCR	4
Leidinger P 2016	Caucasian	blood	74	NA	20	NA	HC	I-III	miR-720, miR-29c, miR-199a, miR-378a,let-7f	91.0	98.0	U24,U48	qRT-PCR	4
Wang WZ 2016	Asian	tissue	15	57	16	58	HC	I-IV	miR-182, miR-10a, miR-301b, miR-1244, miR-301a, miR-135b, miR-224, miR-21	93.3	93.8	miR-16	qRT-PCR	4
Wang WZ 2016	Asian	serum	54	NA	15	NA	HC	I-IV	miR-1244	81.5	80	miR-39	qRT-PCR	4
Kim JL O 2015	Caucasian	BAL	21	70	10	59	HC,BPD	I-II	miR-21, miR-143, miR-155, miR-210, miR-373	85.7	100	U6	qRT-PCR	4
Wang C 2015	Asian	serum	19	61.8	19	62.1	HC	I-IV	miR-483, miR-193a, miR-25, miR-214, miR-7	100	84	let-7d/g/i	qRT-PCR	4
Li WS 2015	Asian	plasma	11	59	11	55	HC	I-III	mir-486	90.9	81.8	miR-39, U44	qRT-PCR	4
Wang C 2015	Asian	serum	63	61.9	63	59.7	HC	I-IV	miR-483, miR-193a, miR-25, miR-214, miR-7	89.0	68.0	let-7d/g/i	qRT-PCR	4
Wang C 2015	Caucasian	serum	108	67.2	56	63.7	BPD	I-IV	miR-483, miR-193a, miR-25, miR-214, miR-7	95.0	95.0	let-7d/g/i	qRT-PCR	4
Wang C 2015	Caucasian	serum	108	67.2	48	58.5	HC	I-IV	miR-483, miR-193a, miR-25, miR-214, miR-7	95.0	84.0	let-7d/g/i	qRT-PCR	4
Nadal E 2015	Caucasian	serum	70	67.5	22	67.0	HC,BPD	I-III	miR-141, miR-200b, miR-193b	96.0	95.0	U6	qRT-PCR	2
Nadal E 2015	Caucasian	serum	84	65.5	23	60.0	HC,BPD	I-III	miR-141, miR-200b, miR-193b	97.0	96.0	U6	qRT-PCR	2
Guo WG 2015	Asian	plasma	126	NA	50	NA	HC	I-IV	mir-204	76.0	82.0	U6	qRT-PCR	4
Ma J 2015	Caucasian, African	PBMC	84	64.1	69	62.4	BPD	I-IV	miR-19b, miR-29b	72.6	82.6	miR-423-3p	qRT-PCR	2
Li L 2015	Asian	serum	36	56.0	30	58.0	HC,BPD	I-IV	miR-148a, miR-148b, miR-152	72.2	90.0	U6	qRT-PCR	4
Zhang XL 2015	Asian	tissue	125	61.0	125	61.0	HC	I-IV	miR-141	64.8	64.8	miR-191, miR-103	qRT-PCR	3
Zhao W 2015	Asian	serum	80	57.6	60	55.4	HC	NA	miR-21	73.8	71.7	U6	qRT-PCR	4
Wang RJ 2015	Asian	serum	70	64.4	70	63.7	HC	NA	miR-145	92.8	61.4	miR-39	qRT-PCR	3
Yang JS 2015	Asian	serum	152	NA	300	NA	HC	I-IV	miR-152, miR-148a, miR-148b, miR-21	96.0	91.0	U6	qRT-PCR	3
Xing LX 2015	Caucasian	sputum	67	66.4	69	64.9	BPD	I-II	miR-21, miR-31, miR-210	82.1	88.4	U6, miR-16	qRT-PCR	4
Liu CM 2015	Asian	Pleural effusion	61	53.8	70	54.4	BPD	NA	miR-192	61.3	79.5	U6	qRT-PCR	2
Dou HL 2015	Asian	plasma	120	63.2	360	NA	HC	I-IV	miR-152	86.0	81.3	U6	digital PCR	4
Yang YL 2015	Asian	PBMC	74	62.5	52	61.8	HC	I-IV	miR-10b	86.5	76.9	miR-16	qRT-PCR	3
Li N 2014	Caucasian, African	sputum	35	68.9	40	65.7	HC	I	miR-31, miR-210	65.7	85.0	NA	qRT-PCR	4
Zhu W 2014	Asian	serum	70	59.0	48	NA	HC	I-IV	miR-429	54.3	81.2	U6,U48	qRT-PCR	4
LI M 2014	Asian	serum	514	NA	54	NA	HC	I-IV	miR-499	73.7	92.7	miR-39	qRT-PCR	3
Ulivi P 2013	Caucasian	blood	86	68.0	24	65.0	HC	I-II	miR-328	70.0	83.0	U38B,U58A	qRT-PCR	4
Bediaga 2013	Caucasian	tissue	45	66.4	45	66.4	HC	I-IV	8 miRNAs_ _^b^	100	97.8	4miRNAs_ _^c^	qRT-PCR	3
Bediaga 2013	Caucasian	tissue	47	67.8	47	67.8	HC	I-IV	8 miRNAs_ _^b^	97.5	96.3	4miRNAs_ _^c^	qRT-PCR	3
Bediaga 2013	Caucasian	tissue	22	68.4	22	68.4	HC	I-IV	8 miRNAs_ _^b^	100	95.0	4miRNAs_ _^c^	qRT-PCR	3
Anjuman 2013	Caucasian, African	sputum	39	65.6	42	62.3	BPD	I	miR-210, miR-31	61.5	90.5	U6	qRT-PCR	4
Tang DF 2013	Asian	plasma	62	64.8	60	66.0	HC	I-III	miR-21, miR-145, miR-155	69.4	78.3	U6	qRT-PCR	1
Tang DF 2013	Asian	plasma	34	65.2	32	66.4	HC	I-III	miR-21, miR-145, miR-155	76.5	81.3	U6	qRT-PCR	1
Mozzoni 2013	Caucasian	plasma	54	69.1	46	64.1	BPD	I-III	miR-21, miR-486	87.0	86.5	miR-16	qRT-PCR	4
ZENG XL 2013	Asian	PBMC	64	58.9	26	54.4	HC	I-IV	miR-143	75.0	92.3	U6	qRT-PCR	4
Yang XQ 2013	Asian	sputum	24	60.5	24	57.8	BPD	I-IV	let-7a	87.5	83.3	U6	digital PCR	4
Ma J 2013	Caucasian, African	plasma	36	66.7	38	64.6	HC	I	miR-21, miR-335	71.8	80.6	NA	qRT-PCR	4
Cazzoli R 2013	Caucasian, African	plasma	50	66.1	30	64.8	BPD	I	miR-151a, miR-30a, miR-200b, miR-629, miR-100, miR-154	96.0	60.0	let7a	qRT-PCR	2
Abd-E 2013	African	Serum	65	54.1	37	50.1	HC	I-II	miR-182	100	86.5	SNORD68	qRT-PCR	4
Sanfiorenzo C 2013	Caucasian	plasma	52	65.1	10	68.9	BPD	I-III	miR-152, miR-145, miR-199a, miR-24, miR-20a, miR-25	90.9	83.3	miR-192, miR-16	qRT-PCR	4
Roa Wilson H 2012	Caucasian	sputum	24	68.8	6	44.7	HC	I-II	miR-21, miR-143, miR-155, miR-210, miR-372	83.3	100	U6	qRT-PCR	4
Li GJ 2012	Asian	plasma	16	NA	14	NA	BPD	I	miR-494, miR-22, miR-200b	85.3	94.5	18S	qRT-PCR	4
Ma YX 2012	Asian	serum	193	NA	110	NA	HC	I-IV	miR-125b	78.2	66.4	NA	qRT-PCR	4
Hennessey P 2012	Caucasian, African	serum	55	68.2	75	65.7	HC	I-IV	miR-15b, miR-27b	100	84.0	miR-16	qRT-PCR	4
ZengXL 2012	Asian	PBMC	34	NA	26	54.4	HC	I-IV	miR-150	87.5	69.2	U6	qRT-PCR	4
Zhao M 2012	Asian	tissue	55	NA	55	NA	HC	I-IV	miR-29a	49.1	85.5	U6	qRT-PCR	3
Shen J 2011	Caucasian, African	plasma	34	68.0	29	66.0	HC	I-IV	miR-21, miR-126, miR-210, miR-486	91.7	96.6	miR-16	qRT-PCR	1
Jeong H 2011	Asian	blood	35	67.0	30	60.0	HC	I-IV	let-7a	90.3	90.3	U6	qRT-PCR	4
Wei J 2011	Asian	plasma	77	59.6	36	56.4	HC	I-IV	miR-21	61.0	83.3	miR-16	qRT-PCR	3
Liu S 2011	Asian	plasma	130	53.1	170	57.5	HC	I-III	miR-126	46.4	90	NA	qRT-PCR	3
Yu L 2010	Caucasian, African	sputum	36	68.2	36	66.7	HC	I	miR-486, miR-21, miR-200b, miR-375	80.6	91.7	U6	qRT-PCR	4
Yu L 2010	Caucasian, African	sputum	64	67.0	58	65.0	HC	I-IV	miR-486, miR-21, miR-200b, miR-375	70.3	80.0	U6	qRT-PCR	3
Xing LX 2010	Caucasian, African	sputum	48	67.5	48	65.9	HC	I	miR-205, miR-210, miR-708	73.0	96.0	U6	qRT-PCR	4
Xing LX 2010	Caucasian, African	sputum	67	68.0	55	65.0	HC	I-IV	miR-205, miR-210, miR-708	72.0	95.0	U6	qRT-PCR	3
Keller Andreas 2009	Caucasian	blood	17	64.2	19	37.9	HC	I-III	24miRNAs_ _^d^	92.5	98.1	NA	qRT-PCR	4

^a^miR-451, miR-1290, miR-636, miR-30c, miR-22-3p, miR-19b, miR-486-5p, miR-20b, miR-93, miR-34b, miR-185, miR-126-5p, miR-93-3p, miR-1274a, miR-142-5p, miR-628-5p, miR-486-3p, miR-425, miR-645, miR-24; ^b^miR-96, miR-450a, miR-183, miR-9, miR-577, Let-7i, miR-27b and miR-34a; ^c^miR-26a, miR-140-5p, miR-195, miR-30b;  ^d^miR-126, miR-423, miR-15a, let-7d, let-7i, miR-22, miR-98, miR-19a, miR-20b, miR-324, miR-574, miR-195, miR-25, let-7e, let-7c, let-7f, let-7a, let-7g, miR-140, miR-339, miR-361, miR-1283, miR-18a, miR-26b; ^*∗*^1: only smokers; 2: smokers and nonsmokers (smoking status was imbalanced between groups); 3: smokers and nonsmokers (smoking status was balanced between groups); 4: unknown smoking status.

N: number; HC: healthy control; BPD: benign pulmonary disease; miR: microRNA; SEN: sensitivity; SPE: specificity; FQDs: fluorescence quantum dots; BAL: bronchoalveolar lavage.

**Table 2:** Subgroup analyses for the selected studies.

**Subgroups**	**No**	**SEN [95**%**CI]**	**SPE [95**%**CI]**	**PLR[95**%**CI]**	**NLR [95**%**CI]**	**DOR[95**%**CI]**	**AUC [95**%**CI]**
MiR profiling							
single	26	0.77[0.71-0.82]	0.80[0.77-0.84]	3.9[3.3-4.7]	0.28[0.22-0.36]	14[10-20]	0.86[0.82-0.88]
multiple	45	0.88[0.85-0.91]	0.91[0.88-0.93]	10.0[7.5-13.3]	0.13[0.10-0.17]	79[50-126]	0.96[0.93-0.97]
Smoking status							
only smokers	4	0.80[0.70-0.87]	0.86[0.77-0.91]	5.6[3.2-9.9]	0.23[0.14-0.38]	24[9-66]	0.90[0.87-0.92]
S+NS (imbalanced)_ _^*∗*^	6	0.88[0.74-0.95]	0.90[0.73-0.97]	9.2[3.0-28.2]	0.13[0.05-0.31]	71[14-360]	0.95[0.93-0.97]
S+NS (balanced) _ _^*∗*^	18	0.83[0.74-0.90]	0.86[0.80-0.90]	5.9[3.9-8.8]	0.19[0.12-0.32]	30[13-69]	0.91[0.88-0.93]
unknown status	43	0.86[0.82-0.89]	0.88[0.85-0.91]	7.3[5.7-9.4]	0.16[0.12-0.21]	46[30-70]	0.93[0.91-0.95]
Specimen							
plasma	22	0.82[0.76-0.87]	0.87[0.83-0.90]	6.3[4.6-8.5]	0.20[0.15-0.28]	31[18-52]	0.92[0.89-0.94]
serum	19	0.91[0.86-0.95]	0.85[0.79-0.89]	6.1[4.3-8.5]	0.10[0.06-0.17]	60[28-128]	0.94[0.91-0.95]
Whole blood/blood cell	9	0.84[0.78-0.89]	0.92[0.80-0.97]	10.9[3.9-30.3]	0.17[0.11-0.26]	64[17-234]	0.92[0.89-0.94]
not blood	21	0.80[0.72-0.86]	0.89[0.85-0.93]	7.5[4.9-11.7]	0.22[0.16-0.32]	34[16-71]	0.92[0.89-0.94]
Ethnicity							
Asian	41	0.82[0.77-0.85]	0.86[0.82-0.88]	5.7[4.5-7.2]	0.21[0.17-0.27]	27[18-40]	0.91[0.88-0.93]
Caucasian	18	0.91[0.86-0.95]	0.92[0.87-0.96]	12[7.0-20.4]	0.09[0.06-0.15]	127[54-302]	0.97[0.95-0.98]
Caucasian/African	12	0.85[0.72-0.93]	0.87[0.81-0.91]	6.6[4.6-9.4]	0.17[0.09-0.33]	39[17-88]	0.92[0.89-0.94]
Control-type							
BPD	13	0.84[0.77-0.89]	0.84[0.80-0.88]	5.3[4.1-6.8]	0.19[0.13-0.28]	27[16-46]	0.90[0.87-0.92]
HC	50	0.86[0.82-0.89]	0.88[0.85-0.91]	7.4[5.7-9.5]	0.16[0.12-0.21]	47[30-74]	0.94[0.91-0.95]
BPD, HC	8	0.81[0.67-0.90]	0.91[0.79-0.96]	8.8[3.4-22.9]	0.21[0.11-0.40]	42[9-187]	0.93[0.90-0.95]
Stage							
I-II	18	0.84[0.78-0.89]	0.90[0.86-0.93]	8.3[5.8-11.9]	0.17[0.12-0.25]	48[27-87]	0.94[0.91-0.96]
I-IV	50	0.86[0.82-0.89]	0.88[0.84-0.90]	6.5[5.4-8.7]	0.16[0.13-0.22]	42[27-66]	0.93[0.90-0.95]
No. of cases							
small	25	0.88[0.82-0.92]	0.91[0.88-0.94]	10.0[7.1-14.2]	0.14[0.09-0.21]	74[38-143]	0.95[0.93-0.97]
large	46	0.84[0.79-0.87]	0.86[0.82-0.88]	5.8[4.6-7.2]	0.19[0.15-0.24]	31[20-46]	0.91[0.89-0.94]
MiR-210	12	0.77[0.72-0.81]	0.93[0.88-0.96]	11.0[6.2-19.4]	0.25[0.20-0.31]	44[22-87]	0.91[0.88-0.93]
MiR-21	16	0.82[0.77-0.86]	0.87[0.84-0.89]	6.3[5.0-8.1]	0.21[0.15-0.28]	31[19-50]	0.91[0.88-0.93]

No: the number of the studies; HC: healthy control; BPD: benign pulmonary disease; SEN: sensitivity; SPE: specificity; PLR: positive likelihood ratio; NLR:negative likelihood ratio; DOR: diagnostic odds ratio; AUC:area under the curve; no. of case: small (<50) and large (≥50).

^*∗*^ S: smokers; NS: nonsmokers; imbalanced: the smoking status was imbalanced between groups; balanced: the smoking status was balanced between groups.

## Abbreviations

miRNA: MicroRNA
NSCLC: Non-small cell lung cancer
SEN: Sensitivity
SPE: Specificity
SROC: Summary receiver operating characteristic
AUC: The area under the SROC curve
mRNA: Messenger RNA
3′-UTR: 3′-untranslated region
BPD: Benign pulmonary disease
HC: Healthy Control
CNKI: Chinese national knowledge infrastructure
PLR: Positive likelihood ratio
NLR: Negative likelihood ratio
DOR: Diagnostic odds ratio
TP: True positive
FP: False positive
FN: False negative
TN: True negative
qRT-PCR: Quantitative real-time polymerase chain reaction
PBMC: Peripheral blood mononuclear cells
PRISMA: Preferred Reporting Items for Systematic Reviews and Meta-Analyses
BAL: Bronchoalveolar lavage.
